# Development of a Monte Carlo-wave model to simulate time domain diffuse correlation spectroscopy measurements from first principles

**DOI:** 10.1117/1.JBO.27.8.083009

**Published:** 2022-02-23

**Authors:** Xiaojun Cheng, Hui Chen, Edbert J. Sie, Francesco Marsili, David A. Boas

**Affiliations:** a Boston University, Neurophotonics Center, Department of Biomedical Engineering, Boston, Massachusetts, United States; b Meta Platforms Inc., Reality Labs Research, Menlo Park, California, United States

**Keywords:** time-domain diffuse correlation spectroscopy, blood flow, noise model, Monte Carlo, wave propagation

## Abstract

**Significance:**

Diffuse correlation spectroscopy (DCS) is an optical technique that measures blood flow non-invasively and continuously. The time-domain (TD) variant of DCS, namely, TD-DCS has demonstrated a potential to improve brain depth sensitivity and to distinguish superficial from deeper blood flow by utilizing pulsed laser sources and a gating strategy to select photons with different pathlengths within the scattering tissue using a single source–detector separation. A quantitative tool to predict the performance of TD-DCS that can be compared with traditional continuous wave DCS (CW-DCS) currently does not exist but is crucial to provide guidance for the continued development and application of these DCS systems.

**Aims:**

We aim to establish a model to simulate TD-DCS measurements from first principles, which enables analysis of the impact of measurement noise that can be utilized to quantify the performance for any particular TD-DCS system and measurement geometry.

**Approach:**

We have integrated the Monte Carlo simulation describing photon scattering in biological tissue with the wave model that calculates the speckle intensity fluctuations due to tissue dynamics to simulate TD-DCS measurements from first principles.

**Results:**

Our model is capable of simulating photon counts received at the detector as a function of time for both CW-DCS and TD-DCS measurements. The effects of the laser coherence, instrument response function, detector gate delay, gate width, intrinsic noise arising from speckle statistics, and shot noise are incorporated in the model. We have demonstrated the ability of our model to simulate TD-DCS measurements under different conditions, and the use of our model to compare the performance of TD-DCS and CW-DCS under a few typical measurement conditions.

**Conclusion:**

We have established a Monte Carlo-Wave model that is capable of simulating CW-DCS and TD-DCS measurements from first principles. In our exploration of the parameter space, we could not find realistic measurement conditions under which TD-DCS outperformed CW-DCS. However, the parameter space for the optimization of the contrast to noise ratio of TD-DCS is large and complex, so our results do not imply that TD-DCS cannot indeed outperform CW-DCS under different conditions. We made our code available publicly for others in the field to find use cases favorable to TD-DCS. TD-DCS also provides a promising way to measure deep brain tissue dynamics using a short source–detector separation, which will benefit the development of technologies including high density DCS systems and image reconstruction using a limited number of source–detector pairs.

## 1 Introduction

Cerebral blood flow (CBF) is an important indicator of brain function and health.^1^^–^^6^ Diffuse correlation spectroscopy (DCS) serves as a major optical technique that is capable of measuring blood flow non-invasively and continuously.^7^^–^^11^ In DCS, coherent light is incident on the surface of the scattering medium, and the re-emitted scattered light is collected by a detector at a certain distance away from the source position, typically in the range of 10 to 30 mm. Dynamics in the tissue, mainly arising from blood flow induces phase variations of the scattered light that alters the interference pattern of the partial waves of the re-emitted light, namely, the speckle pattern, thus causing light intensity fluctuations with time at the detector. It is common to quantify these temporal fluctuations using a temporal intensity autocorrelation function. The decay rate of this autocorrelation function provides a measure of the blood flow index. Numerical and theoretical tools have been developed to guide interpretations of experimental measurements. For traditional continuous-wave DCS (CW-DCS), the analytical expressions of the autocorrelation functions have been well established in diffusion theory,^7^^,^^12^^,^^13^ and Monte Carlo simulations have been developed to numerically obtain the field autocorrelation function for inhomogeneous media.^8^^,^^14^ Together with the noise model obtained analytically,^15^ the performance of a CW-DCS system for a particular measurement geometry can be theoretically predicted.

Recently, a time-domain variant of DCS (TD-DCS) has been developed, which utilizes a pulsed laser and a gating strategy to select photons detected at different arrival times.^16^^–^^20^ This technology has the potential to differentiate blood flow information from different layers, such as the skull and the brain tissue using a single source–detector separation. The theoretical basis for TD-DCS has been established taking into account the effects of the instrument response function (IRF), which includes the incident pulse shape and the broadening of the pulse arising from the instrument, and the coherence length of the light source on the shape of the intensity autocorrelation function.^18^ Ideally, a narrower width of the IRF will result in better specificity to different photon pathlengths. However, the width of IRF is fundamentally limited by the coherence length, and a narrow pulse in time will result in a short coherence length, which will degrade signal to noise ratio (SNR) in TD-DCS measurements.^18^ A pioneering study on the performance of TD-DCS has been conducted assuming the same noise model^15^ that is developed for CW-DCS.^21^ A proper noise model that quantifies the performance of TD-DCS measurements in terms of SNR and contrast to noise ratio (CNR) for any IRF, coherence length, measurement geometry, and brain activation pattern is still lacking.

We have constructed a numerical model which is capable of simulating both CW- and TD-DCS measurements from first principles. We have integrated Monte Carlo simulations with wave calculations to obtain the intensity fluctuations induced by tissue dynamics. The speckle intensity is converted to photon counts within each bin time mimicking the experimental measurements. The numerical results are compared with existing theoretical expressions of the average intensity autocorrelation function for CW-DCS^14^ and TD-DCS.^18^ The simulated noise is also validated with the existing noise model for CW-DCS.^15^ We have demonstrated the value of using our model to predict the performance for any TD-DCS systems and measurement geometry in terms of CNR due to brain activation. We have found that for a given set of parameters, brain geometry, and the IRF profile utilized in this paper, CW-DCS at a large source–detector separation (30 mm) out-performs TD-DCS. We can increase the detected photon flux per bin time of TD-DCS at a small source–detector separation (10 mm) by increasing the gate width at the cost of reduced specifity to a particular photon pathlength or depth to achieve a performance comparable to CW-DCS at a medium source–detector separation (20 mm). We could not find realistic measurement conditions under which TD-DCS outperformed CW-DCS. However, since the performance of TD-DCS is highly dependent on the IRF profile^21^ and the other measurement parameters, the quantitative results predicted in this paper can be different for researchers using different input parameters in our model. The performance for a given TD-DCS system can be easily calculated by changing the parameters of the numerical model, which is made publicly available.^22^ TD-DCS is also capable of differentiating blood flow at different depths using a single source–detector separation. This model provides guidance for the experimental development of novel TD-DCS systems.

## 2 Method

### 2.1 Theory

**Fig. 1:**
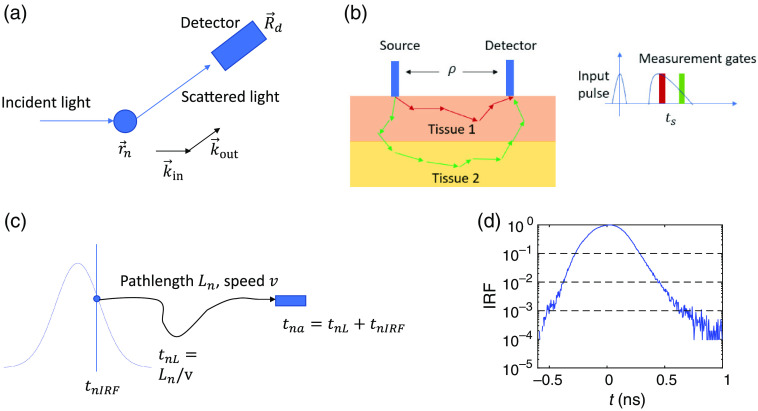
(a) Illustration of the single scattering event and the parameters used in Eq. (1). (b) The geometry of the Monte Carlo simulation used in this manuscript and the illustration of the gating strategy for TD-DCS. The size of the detector is 2 mm, the thickness of the first layer or first tissue type is 15 mm, the reduced scattering coefficient is $\mu_{s^{\prime }}=1\;{\mathrm{mm}}^{-1}$ and absorption coefficient $\mu_{a}=0.01\;{\mathrm{mm}}^{-1}$. The optical properties $\mu_{s^{\prime }}$ and $\mu_{a}$ are set to be the same for the two layers. The dynamics in the second layer is increased when brain activation is introduced. The source detector separation is set to be ρ=20  mm unless otherwise stated. Measurements at a later gate will be able to select photons with longer pathlengths, thus it is more sensitive to dynamics at deeper depths. (c) Illustration of the calculation of the photon arrival time for the nth photon as the sum of the IRF time and the transit time within the sample $t_{na}=t_{n\mathrm{RIF}}+t_{nL}$. (d) The IRF profile we have used in this manuscript obtained experimentally. The full width half maximum of the IRF is 0.31 ns. The dashed lines indicate the levels of the intensity of the IRF that corresponds to 0.1, 0.01, and 0.001 of the peak intensity, and the widths that correspond to these levels are 0.56, 0.82, and 1.18 ns, respectively.

DCS quantifies the dynamics of the sample by calculating the autocorrelation function of the speckle intensity fluctuations of light reemitted from tissue. For a single scattering event shown in Fig. 1(a), the electric field measured at the detector is

$$\overset{\to }{E}\propto {\overset{\to }{E}}_{0}\overset{N}{\underset{n=1}{\sum }}\;\exp(i{\overset{\to }{k}}_{\text{in}}\cdot {\overset{\to }{r}}_{n})\exp(i{\overset{\to }{k}}_{\text{out}}\cdot ({\overset{\to }{R}}_{d}-{\overset{\to }{r}}_{n}))\;\propto {\overset{\to }{E}}_{0}\;\exp(i{\overset{\to }{k}}_{\text{out}}\cdot {\overset{\to }{R}}_{d})\overset{N}{\underset{n=1}{\sum }}\;\exp(-i{\overset{\to }{q}}_{n}\cdot {\overset{\to }{r}}_{n}).$$ (1)

Here, $\overset{\to }{E}$ is the electric field reaching the detector located at ${\vec{R}}_{d}$; ${\vec{r}}_{n}$ is the position of the n’th scatterer; N is the total number of the scatterers; ${\vec{E}}_{0}$ is the incident field; and ${\overset{\to }{q}}_{n}={\overset{\to }{k}}_{\text{out}}-{\overset{\to }{k}}_{\text{in}}$ is the momentum transfer, where ${\overset{\to }{k}}_{\text{out}}$ and ${\overset{\to }{k}}_{\text{in}}$ are the output and incident wave vectors, respectively. We only consider elastic scattering such that $|{\overset{\to }{k}}_{\text{out}}|=|{\overset{\to }{k}}_{\text{in}}|=k$.

For the case of multiple light scattering, Eq. (1) is revised as

$$\vec{E}\propto \sum_{n=1}^{N}\sum_{s=1}^{N_{sn}}\exp(-i{\vec{q}}_{ns}\cdot {\vec{r}}_{ns}).$$ (2)

Here, s denotes the s’th scattering event, and $N_{sn}$ is the total number of scattering events for the n’th component of the wave, or the n’th photon in our Monte Carlo simulations as will be described in Sec. 2.2. The field autocorrelation function is calculated from $g_{1}(\tau )=\langle E^{*}(t)E(t+\tau )\rangle /\langle {\left|E(t)\right|}^{2}\rangle$. For a semi-infinite medium, the analytical form of $g_{1}$ for CW-DCS system has been calculated as^7^^,^^9^

$$g_{1}(\rho ,\tau )=\frac{3C\mu_{s^{\prime }}}{4\pi }[\frac{\exp(Kr_{1})}{r_{1}}-\frac{\exp(Kr_{2})}{r_{2}}].$$ (3)

Here, $K^{2}=3\mu_{a}\mu_{s^{\prime }}+6\mu_{s^{\prime }}^{2}k_{0}^{2}D_{B}\tau$, ρ is the source–detector distance, $r_{1}={\left(\rho^{2}+z_{0}^{2}\right)}^{1/2}$, $r_{2}={\left(\rho^{2}+{\left(z_{0}+2z_{b}\right)}^{2}\right)}^{1/2}$, $z_{0}=1/\mu_{s^{\prime }}$, $z_{b}=(5/3)\mu_{s^{\prime }}$, k=2π/λ, λ is the wavelength, $k_{0}$ is the central wave vector, C is the normalization constant, $D_{B}$ is the diffusion coefficient, and $\mu_{a}$ and $\mu_{s^{\prime }}$ are the absorption and reduced scattering coefficients, respectively. In real time measurements, a simpler expression is preferred for fitting and Eq. (3) can be simplified to a single exponential decay at small τ limit^15^^,^^23^

$$g_{1}(\tau )=\exp(-\tau /\tau_{c}),$$ (4)

where $\tau_{c}$ is the decay time constant that is utilized to quantify the tissue dynamics. Experimentally, instead of $g_{1}(\tau )$, the intensity correlation function $g_{2}(\tau )=\langle I(t)I(t+\tau )\rangle /{\left\langle |I(t)|\right\rangle }^{2}$ is measured, and $g_{2}(\tau )$ is related to $g_{1}(\tau )$ via the Siegert relation^24^

$$g_{2}(\tau )=1+\beta g_{1}{\left(\tau \right)}^{2},$$ (5)

with β=1 indicating complete coherence of the detected photons, and β<1 accounting for loss of temporal coherence and/or detection of multiple modes such as the two polarization states of the electromagnetic field. The effect of the coherence length $l_{c}$ has been estimated as^25^

$$g_{2}(\tau )=1+\int_{0}^{\infty }dL\int_{0}^{\infty }dL^{\prime }P(L)P(L^{\prime })g_{1}(L,\tau )g_{1}(L^{\prime },\tau )e^{-16{\left[(L-L^{\prime })/(\pi l_{c})\right]}^{2}}.$$ (6)

Here, P(L) is the photon pathlength distribution. It is noteworthy that this relation is only valid when the incident light spectrum is a Gaussian function. For a semi-infinite highly scattering medium relevant for human brain measurements, P(L) can be calculated as^26^^,^^27^

$$P(L)=\frac{vS}{{\left(4\pi DL/v\right)}^{3/2}}\;\exp(-\mu_{a}L)[\exp(-\frac{z_{-}^{2}}{4DL})-\exp(-\frac{z_{+}^{2}}{4DL})].$$ (7)

Here, v is the speed of light in the sample, $z_{-}^{2}=z_{0}^{2}+\rho^{2}$, $z_{+}^{2}={\left(z_{0}+2z_{b}\right)}^{2}+\rho^{2}$, $z_{0}=1/\mu_{s^{\prime }}$, $z_{b}=2D(1+R_{\mathrm{eff}})/(1-R_{\mathrm{eff}})$, $R_{\mathrm{eff}}$ the boundary reflectivity, $D=1/[3(\mu_{a}+\mu_{s^{\prime }})]$, $\mu_{a}$ the absorption coefficient, $\mu_{s^{\prime }}$ the reduced scattering coefficient, and S the normalization factor such that $\int_{0}^{\infty }P(L)dL=1$. Here, $g_{1}(L,\tau )$ is the pathlength-dependent normalized field temporal autocorrelation function

$$g_{1}(L,\tau )=\exp(-2\mu_{s^{\prime }}D_{B}k_{0}^{2}L\tau ),$$ (8)

At τ=0, $g_{1}(L,0)=1$, which provides the relation between β and $l_{c}$ in Eq. (6).

The noise model for CW-DCS systems has been established analytically.^15^ The standard deviation of $\left(g_{2}(\tau )-1)\right)$, σ(τ), at each time delay τ is

$$\sigma (\tau )=\sqrt{T_{b}/T}[\beta^{2}\frac{(1+e^{-2\Gamma T_{b}})(1+e^{-2\Gamma \tau })+2m(1-e^{-2\Gamma T_{b}})e^{-2\Gamma \tau }}{1-e^{-2\Gamma T_{b}}}\;+2{\left\langle n\right\rangle }^{-1}\beta (1+e^{-2\Gamma \tau })+{\left\langle n\right\rangle }^{-2}(1+\beta e^{-\Gamma \tau }){]}^{1/2}.$$ (9)

Here, $T_{b}$ is the bin time, m is the bin index, T is the measurement time window, ⟨n⟩ is the average number of photons in a bin time $T_{b}$, $g_{2}(\tau )-1=\beta e^{-2\Gamma \tau }$, where $\Gamma =1/\tau_{c}$.

For TD-DCS, a gating strategy is utilized to select a portion of the photons that arrive at the detector. For a gate delay time $t_{s}$ and gate width $t_{w}$, only the photons that arrive within a measurement window are selected to calculate the electric field in Eq. (2), as shown in Fig. 1(b). Ideally, if both the widths of the IRF and the gate width are infinitely small, the gating strategy will only select photons with a single pathlength $L=t_{s}*v$, and $g_{1}(\tau )$ is reduced to $g_{1}(L=t_{s}*v,\tau )$ in Eq. (8), where v is the speed of the light in the tissue. However, both the IRF and the gate width will increase the width of the distribution of the photon pathlengths detected within a measurement window. The expression of $g_{2}(\tau )$ for TD-DCS has been analytically obtained and the effect of the coherence length $l_{c}$ and IRF are taken into account.^18^ At $t_{s}$, the gate delay time dependent intensity autocorrelation function $g_{2}(t_{s},\tau )$ is

$$g_{2}(t_{s},\tau )=1+\int_{0}^{\infty }dL\int_{0}^{\infty }dL^{\prime }P(L)I_{p}(t_{s}-L/v)P(L^{\prime })\;I_{p}(t_{s}-L^{\prime }/v)g_{1L}(L,\tau )g_{1L}(L^{\prime },\tau )e^{-16{\left[(L-L^{\prime })/(\pi l_{c})\right]}^{2}}.$$ (10)

Here the effective pathlength distribution $P(t_{s},L)=P(L)I_{p}(t_{s}-L/v)$ is the pathlength distribution of photon trajectories measured at $t_{s}$, where the normalized IRF profile is denoted as $I_{p}(t)$. The definition of coherence length is shown in Sec. 2.2, Step 4. It is noteworthy that the definition of the coherence length can be different in other studies.^18^^,^^25^

### 2.2 Monte Carlo Wave Model

We have established a numerical recipe to simulate photon counts received at the detector as a function of time for both CW-DCS and TD-DCS measurements from first principles. The steps needed to construct the model are explained below.

Step 1: Simulate photon migration within a scattering medium using Monte-Carlo simulations. The code for the Monte Carlo simulation is a derivation of that used in previous publications.^14^^,^^23^^,^^28^ In the Monte Carlo simulation, we specify the source–detector separation and the optical properties of the sample including the scattering coefficient $\mu_{s}$ and anisotropy factor g in different tissue types. We have used a two-layer sample geometry denoted as two tissue types in the Monte Carlo code as shown in Fig. 1(b). Here we have set g=0 and $\mu_{s}=\mu_{s^{\prime }}=1\;{\mathrm{mm}}^{-1}$ for both tissue types in this study, but these can be easily configured to be different by researchers for future work. In biological tissue, the value of g obtained experimentally is typically above 0.9.^29^ But in the diffusive regime as we are interested, the DCS results are only governed by the reduced scattering coefficient as indicated in Eq. (3). Thus, we can implement isotropic scattering with g=0 and use a smaller $\mu_{s}$ by assigning $\mu_{s}=\mu_{s^{\prime }}$ in the Monte Carlo simulation to improve computational efficiency, which is equivalent to g=0.9, $\mu_{s^{\prime }}=\mu_{s}(1-g)$. The effect of absorption will be taken into account at a later stage in Step 8. We will assign different blood flow dynamics to the two layers to demonstrate the DCS signal variation induced by blood flow change due to neural activation in the deeper layer. The number of scattering events $N_{sni}$ and pathlength $L_{nti}$ for the n’th photon in the i ti’th tissue type are recorded. The values of other parameters we have used in the model are specified in the caption of Fig. 1(b).
Step 2: Calculate the arrival time $t_{na}$ of the n’th photon. We set the time at the peak of the IRF to be t=0. The transit time that a photon spent within the medium is $t_{nL}=L_{n}/v$, where v=c/n is the speed of light within the medium, c=300  mm/ns is the speed of the light in vacuum and the refractive index of the tissue is set to be n=1.33. The IRF time $t_{n\mathrm{IRF}}$ is randomly drawn from the probability density distribution determined by the IRF profile, by obtaining the time at which a random number drawn within the interval [0 1] matches the cumulative probability. This takes into account the effect of the IRF on the distribution of the photon pathlengths detected within a specific measurement window. The arrival time of the photon is $t_{na}=t_{nL}+t_{n\mathrm{IRF}}$. In this paper, we have used an experimentally measured IRF as shown in Fig. 1(d). In the experiment, a PicoQuant VisIR laser at 765 nm was used as the source with a PPG512 to trim the pulse, and a PicoQuant PMA42 photomultiplier was used as the detector to reduce the long tail. Any IRF profile obtained experimentally can be utilized in this model to predict the performance for a particular TD-DCS system.
Step 3: Select photons that fall within the specified measurement gate. For a gate delay time $t_{s}$ with a gate width $t_{w}$, only the photons with $t_{na}$ that fall within the time span $\left[t_{s}-t_{w}/2,t_{s}+t_{w}/2\right]$ are selected for the following calculations. It is noteworthy that if both the width of the IRF and the gate width $t_{w}$ are infinitely small, the photons with a single pathlength $L_{n}=t_{s}*v$ are selected, which is the ideal case described by Eq. (8), but is generally not achievable experimentally.
Step 4: For each photon, assign the spectrum of the input light that corresponds to a particular coherence length $l_{c}$. Here we have utilized a Gaussian spectrum, and the relation between the Gaussian spectrum in real and k space is related via
  $$S(k)=e^{-{\left(k-k_{0}\right)}^{2}/(2k_{c}^{2})},\;S(L)=e^{-L^{2}/(2l_{c}^{2})},\;k_{c}^{2}=\pi^{2}/(4l_{c}^{2}).$$ (11)

Here, we have defined the coherence length $l_{c}$ as the width of the Gaussian. It is noteworthy that the definition of the coherence length can be different in other studies. We have combined the photon description of light with the wave description. In principle, we cannot assign a wavelength to a photon. However, each photon can be considered as a light trajectory for wave propagation, which allows us to calculate the accumulated phase along a light path for different wavelengths. In our model, any spectrum shape S(k) measured experimentally can be incorporated, which is one advantage of our numerical model over existing theoretical models,^18^^,^^25^ where a Gaussian spectrum profile is always assumed.

Step 5: Assign an initial phase randomly drawn within [0  2π] for each photon trajectory $\phi_{n}$.
Step 6: Obtain the displacements of the scatterers as functions of time. Here we denote the displacement of the scatterer in the s’th scattering event of the n’th photon trajectory $\Delta {\vec{r}}_{ns}(t)$. The displacement is obtained from a second Monte Carlo simulation of random particle motion with a diffusion coefficient $D_{B}$. The displacement of a scatterer $\Delta {\vec{r}}_{ns}(t)=(\vec{r}(t)-\vec{r}(0){)}_{ns}$ can be calculated from the evolution of $\vec{r}(t)=(x(t),y(t),z(t))$
  $$x(t+\Delta t)=x(t)+N(0,2D_{B}\Delta t),\;y(t+\Delta t)=y(t)+N(0,2D_{B}\Delta t),\;z(t+\Delta t)=z(t)+N(0,2D_{B}\Delta t).$$ (12)

Here, $N(0,2D_{B}\Delta t)$ is a normal distribution with mean 0 and variance $2D_{B}\Delta t$. It is noteworthy that the motion of the scatterers or red blood cells is in general a combination of convective flow and shear induced diffusion. It has been experimentally observed and numerically demonstrated that the DCS signal is dominated by the diffusive behavior of RBCs red blood cells (RBCs).^14^^,^^30^^,^^31^ Thus, we only model the diffusive motion of RBCs as in Eq. (12). The diffusion coefficient at the baseline, i.e. without brain activation, is set to be $D_{B}=1*10^{-6}\;{\mathrm{mm}}^{2}/s$ in the model, which provides a decay time constant close to experimental measurements.^9^^,^^23^

Step 7: Calculate the momentum transfer ${\overset{\to }{q}}_{\mathrm{nis}}=({\overset{\to }{k}}_{\text{out}}-{\overset{\to }{k}}_{\mathrm{in}}{)}_{\mathrm{nis}}$ for each scattering event. Here i denotes the i’th wave vector k for the s’th scattering event of the n’th photon. The angle of a scattering event can be sampled from the phase function. However, since we are assuming g=0 where the scattering is isotropic as described in Step 1, we can simply generate unit vectors ${\overset{\to }{n}}_{\text{out}}$ with a random direction and calculate ${\overset{\to }{n}}_{\text{out}}-{\overset{\to }{n}}_{\text{in}}$ using a coordinate space where ${\overset{\to }{n}}_{\text{in}}=(0,0,1)$. The momentum transfer is then ${\vec{q}}_{\mathrm{nis}}=k_{i}*({\vec{n}}_{\mathrm{out}}-{\vec{n}}_{\mathrm{in}}{)}_{\mathrm{ns}}$.
Step 8: At every time point t, obtain the contribution of the n’th photon and i’th k vector to the electric field as
  $$E_{n,i}(t)=\sqrt{S(k)}e^{i(\phi_{n}+k_{i}L_{n})+\underset{s}{\sum }i{\overset{\to }{q}}_{ins}*\overset{\to }{\Delta }r_{ns}(t)}{\sqrt{e^{-\mu_{a}L_{n}}}}^{i(\phi_{n}+k_{i}L_{n})-\underset{s}{\sum }i{\overset{\to }{q}}_{ins}\cdot \overset{\to }{\Delta }r_{ns}(t)}\sqrt{e^{-\mu_{a}L_{n}}}.$$ (13)

It is noteworthy that if $\mu_{a}$ is different in different tissue types, $\mu_{a}L_{n}=\underset{ti}{\sum }\mu_{ati}L_{nti}$, where $\mu_{ati}$ and $L_{nti}$ are the absorption coefficient and photon pathlength in the ti’th tissue type

Step 9: Calculate the intensity fluctuations I(t). This is calculated by summing over the contribution from all the photon trajectories and wavelengths
  $$I(t)=\underset{i}{\sum }{\left|\underset{n}{\sum }E_{n,i}(t)\right|}^{2}.$$ (14)

**Fig. 2:**
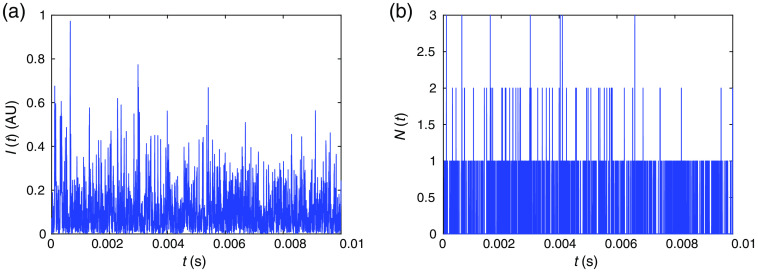
Example of (a) I(t) and (b) N(t) generated from the Monte Carlo wave model assuming the average photon flux is 100,000  photons/s. Here (b) is generated from (a) following the procedure in Step 10.

Here we have ignored the term $e^{i\omega t}$, where ω is the angular frequency of the light, and we have performed an incoherent sum over the k vectors since typically the measurement time is long enough such that light waves with different angular frequencies or wavelengths do not interfere. An example of the simulated intensity temporal fluctuations I(t) is shown in Fig. 2(a).

Step 10: Convert intensity I(t) to photon counts N(t). If the average photon flux within a bin time Δt is $N_{\text{bin}}$, we then normalize I(t) such that the average value of ${\left\langle I(t_{n})\right\rangle }_{n}=N_{\text{bin}}$, where $I(t_{n})$ is the intensity within the n’th bin time $t_{n}$. Then we perform a Poisson draw for each bin time as, $N(t_{n})=Pois(I(t_{n}))$. This process converts the continuous function I(t) to discrete values as well as incorporates shot noise into the numerically generated photon counts N(t). Other sources of noise can also be added to N(t), including dark noise, but this is out of the scope of this paper as dark noise is usually negligible in experimental systems. An example of the conversion from I(t) in Fig. 2(a) to N(t) is shown in Fig. 2(b). The Poisson draw here is not unique due to the nature of shot noise.
Step 11: Calculate the intensity autocorrelation function $g_{2}(\tau )=\langle N(t)N(t+\tau )\rangle /{\left\langle N(t)\right\rangle }^{2}$. It is noteworthy that if we skip Step 10, I(t) can also be utilized to calculate the intrinsic noise in $g_{2}(\tau )=\langle I(t)I(t+\tau )\rangle /{\left\langle I(t)\right\rangle }^{2}$ that arises from speckle statistics due to a finite measurement time,^32^ which does not include the effect of shot noise.

For CW-DCS simulations, we skip Steps 2 and 3, and use all the recorded photons to calculate the speckle fluctuations utilizing Eqs. (13) and (14). In Sec. 3, we compare our modeling results with existing theoretical predictions described in Sec. 2.1.

## 3 Results

**Fig. 3:**
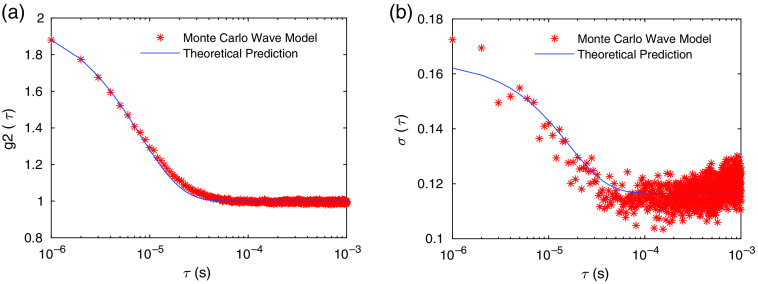
(a) $g_{2}$ obtained from the Monte Carlo wave model averaged over 500 independent simulations compared with the theoretical expression of $g_{2}(\tau )=1+(\exp(-2\tau /\tau_{c}))$. (b) $\sigma (\tau )=\mathrm{std}(g_{2}(\tau )-1)$ obtained from Monte Carlo wave model with 500 independent simulations compared with the theoretical prediction given in Eq. (9). The parameters used are $T_{b}=1\;\mu s$, T=10  ms, β=1, ⟨n⟩=0.1, and $\tau_{c}\approx 32\;\mu s$ obtained from fitting in (a). The total number of photons used in the Monte Carlo simulation is $10^{8}$.

We first simulate CW-DCS measurements with the measurement configuration described in Fig. 1(b). We compare our results with the theoretical predictions of both $g_{2}(\tau )$ in Eq. (4) and the noise model in $g_{2}(\tau )$ and σ(τ) in Eq. (9) as shown in Figs. 3(a) and 3(b). We see that our numerical results are in good agreements with the theoretical predictions. These results are obtained for a single wavelength at 800 nm under the assumption that the coherence length is infinitely long.

**Fig. 4:**
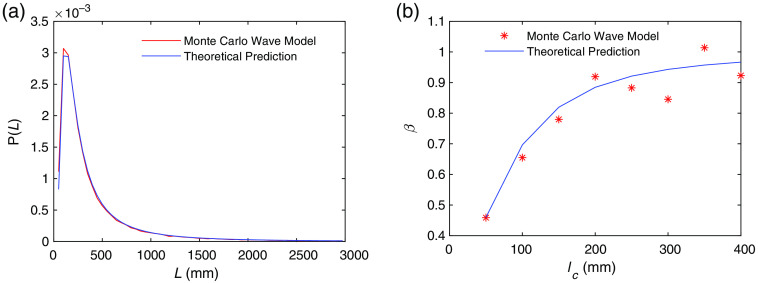
(a) Pathlength distribution of all the photons that arrive at the detector obtained from the Monte Carlo wave model and the theoretical prediction from Eq. (7). The total number of detected photons is $N_{p}=34759$. (b) Comparison of the Monte Carlo wave model prediction for the dependence of β on coherence length with the analytical model in Eq. (9).

Next, we simulate CW-DCS measurements using multiple wavelengths with central wavelength 800 nm to compare with the theoretical predictions of the reduction of β due to loss of coherence described by Eq. (6), by varying the width of the source spectrum (i.e., the coherence length). The pathlength distribution P(L) of all the photons detected at the detector agrees with the theoretical prediction in Eq. (7), as shown in Fig. 4(a). With the same P(L), we have calculated $\beta =g_{2}(0)-1$ as a function of the coherence length $l_{c}$ using our numerical model and compared with the results calculated from the theoretical prediction in Eq. (6). In these calculations, we have used I(t) instead of N(t) to obtain $g_{2}(\tau )$ in Step 11 of Sec. 2.2, since the effect of shot noise is not included in Eq. (6).^25^

**Fig. 5:**
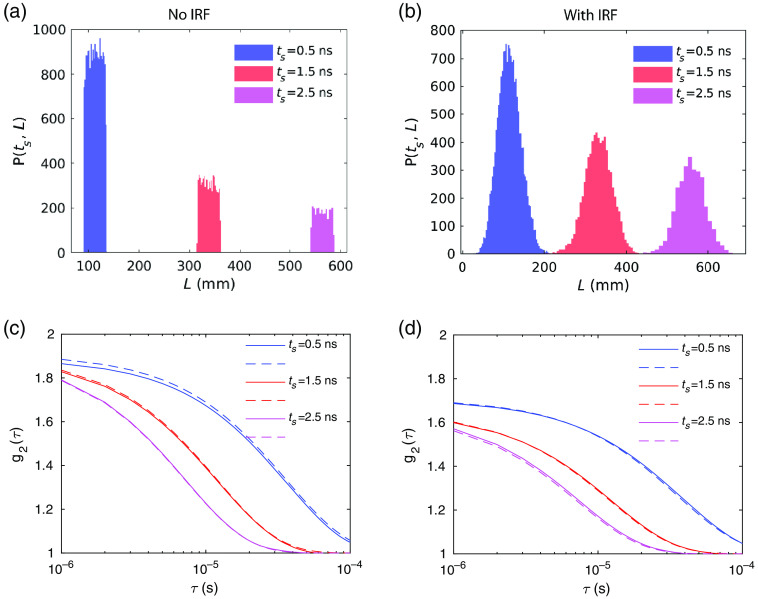
The histogram for the gate delay time dependent pathlength distribution $P(t_{s},L)$ for the case when (a) the effect of IRF is not taken into account and (b) the effect of IRF is taken into account. (c) $g_{2}(\tau )$ obtained from the Monte Carlo wave model (solid lines) and analytical predictions (dashed lines) in Eq. (10) for the case when (c) the effect of IRF is not taken into account and (d) the effect of IRF is taken into account. We have calculated results for $t_{s}=0.5$, 1.5, 2.5 ns, coherence length $l_{c}=90\;\mathrm{mm}$, gate width $t_{w}=0.2\;\mathrm{ns}$, source–detector separation ρ=20  mm. Other parameters are the same as in Fig. 3.

After validating our modeling results with existing theoretical predictions for CW-DCS systems, we now simulate TD-DCS measurements with and without the IRF profile in Fig. 1(d) taken into account, using a coherence length of $l_{c}=90\;\mathrm{mm}$ for the input light source. For the case without the effect of the IRF, the IRF profile is assumed to be infinitely narrow, i.e., $t_{n\mathrm{IRF}}=0$, $t_{na}=t_{nL}=L/v$ in Step 2 of Sec. 2.2. The gate delay time dependent pathlength distributions $P(t_{s},L)$ for $t_{s}=0.5$, 1.5, and 2.5 ns and $g_{2}(\tau )$ with and without the IRF are shown in Figs. 5(a) and 5(b). We see that if the effect of the IRF is ignored, the pathlength distribution $P(t_{s},L)$ is solely determined by the gates including gate delay $t_{s}$ and gate width $t_{w}$. Compared to Fig. 5(a), the pathlength distributions in Fig. 5(b) are broadened and their shapes are determined by the IRF profile in Fig. 1(d). The corresponding results of $g_{2}(\tau )$ are shown in Figs. 5(c) and 5(d). Compared to Fig. 5(c), the values of $\beta =g_{2}(0)-1$ in Fig. 5(d) are smaller due to a broader distribution of pathlengths, as we have expected. To validate the modeling results, we compare the $g_{2}(\tau )$ curves obtained from the Monte Carlo wave model (solid lines) with those obtained from the analytical expression in Eq. (10) (dashed lines) as shown in Figs. 5(c) and 5(d). We see that the modeling and analytical results are in good agreement. It is noteworthy that for Figs. 5(a) and 5(c), we have assumed a finite coherence length with an infinitely small width of the IRF, which is generally not physically realizable. This numerical analysis serves as a sanity check against established concepts.

**Fig. 6:**
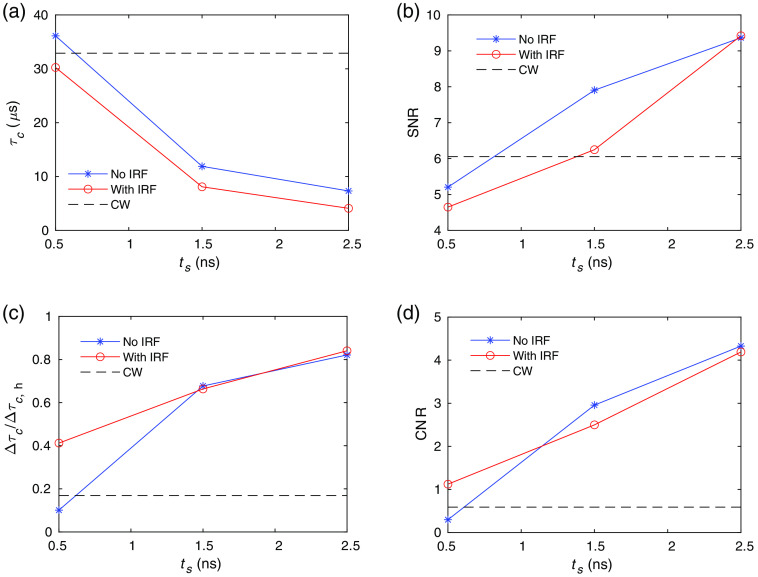
Comparison of the performance of TD-DCS and CW-DCS in the speckle noise limit. Since CW-DCS results do not vary with $t_{s}$, they are denoted as dashed lines with constant y values. (a) $\tau_{c}$, (b) SNR, and (c) the ratio of the contrast for activation in the second layer and activation in the full regime $\Delta \tau_{c}/\Delta \tau_{c,h}$, and (d) CNR for CW-DCS, and TD-DCS with and without the effect of the IRF taken into account. The parameters used here are the same as in Fig. 5.

Next, we perform a noise analysis when only the speckle noise is considered, i.e., in the high photon flux limit. We first obtain $g_{2}(\tau )=\langle I(t)I(t+\tau )\rangle /{\left\langle I(t)\right\rangle }^{2}$ and then obtain $\tau_{c}$ from fitting using Eq. (4). The signal is obtained as the average of the $\tau_{c}$ values, which is denoted as $\tau_{c}$ for simplicity. Noise is obtained from the $\mathrm{std}(\tau_{c})$ by running 100 instances of the Monte Carlo wave model simulations using the baseline sample properties. The SNR is defined as $\mathrm{SNR}=\tau_{c}/\mathrm{std}(\tau_{c})$. To calculate the CNR for estimating changes in blood flow, we produce an activated state by changing the diffusion coefficient from the baseline value $D_{B}=1\times 10^{-6}\;{\mathrm{mm}}^{2}/s$ to $D_{B}=2.25\times 10^{-6}\;{\mathrm{mm}}^{2}/s$ in the second layer (Tissue 2) shown in Fig. 1(b). The value of $\tau_{c^{\prime }}$, which is the average $\tau_{c}$ at the activated states, is obtained from the average of 10 instances of the wave model. The contrast is $\Delta \tau_{c}=\tau_{c}-\tau_{c^{\prime }}$, and the CNR is $\mathrm{CNR}=\Delta \tau_{c}/\mathrm{std}(\tau_{c})$. Here, we have used a large change of the diffusion coefficient to minimize errors in the contrast induced by noise to save computational time, since this paper is mainly focused on demonstrations of the model instead of calculating the realistic contrast due to brain activation. A smaller change of $D_{B}$ can be applied, while more configurations used for averaging are required to obtain both $\tau_{c}$ and $\tau_{c^{\prime }}$. The comparison of the performance of TD-DCS with and without the effect of IRF, and CW-DCS in the speckle noise limit are shown in Fig. 6. The value $\tau_{c}$ decreases with increasing $t_{s}$ in Fig. 6(a) as expected, since photons with longer pathlengths that undergo more scattering events are selected at larger $t_{s}$. The SNR increases with $t_{s}$ as shown in Fig. 6(b). This is because for a fixed measurement time (T=10  ms in this case), more speckle evolutions are averaged at longer $t_{s}$ because of the shorter $\tau_{c}$. To quantify the specificity to brain activation in the second layer in Fig. 1(b), we obtain the contrast with activation only in the second layer, i.e., the diffusion coefficient is increased from baseline value $D_{B}=1\times 10^{-6}\;{\mathrm{mm}}^{2}/s$ to $D_{B}=2.25\times 10^{-6}\;{\mathrm{mm}}^{2}/s$ in the second layer (Tissue 2), and compared with the case when brain activation is applied to the full medium, i.e., the diffusion coefficient is increased from baseline value $D_{B}=1\times 10^{-6}\;{\mathrm{mm}}^{2}/s$ to $D_{B}=2.25\times 10^{-6}\;{\mathrm{mm}}^{2}/s$ in the whole simulation region with its contrast denoted as $\Delta \tau_{c,h}$. We see in Fig. 6(c) that the specificity to brain activation in the second layer, i.e., $\Delta \tau_{c}/\Delta \tau_{c,h}$, increases with increasing $t_{s}$, and that at large $t_{s}$, $\Delta \tau_{c}/\Delta \tau_{c,h}$ is larger for TD-DCS than for CW-DCS. This demonstrates the ability of TD-DCS measurements at larger $t_{s}$ to achieve better sensitivity to tissue dynamics at deeper layers. Thus, TD-DCS can tune the depth specificity by changing the measurement gate delays with a fixed source–detector geometry, which is convenient for technologies such as high-density DCS measurements. Also, CNR is higher for TD-DCS compared to CW-DCS in this high-photon flux, speckle noise limit as shown in Fig. 6(d).

Now, we consider the more realistic case for human brain measurements where the photon flux is very low and the DCS signal is shot noise limited instead of speckle noise limited. The photon flux difference between TD-DCS and CW-DCS measurements needs to be taken into account. We assume the incident photon flux to be 100,000  photons/s for CW-DCS at ρ=20  mm. We then scale the photon flux accordingly by the number of photons detected in a measurement gate in Step 3 for TD-DCS and the total number of the detected photons in Step 1 of the Monte Carlo simulation for CW-DCS, as shown in Table 1. For the low photon fluxes typical of human measurements, long measurement times T are needed to get estimates of $g_{2}(\tau )$ with sufficient quality that can be fit to obtain an estimate of $\tau_{c}$. Running the wave model for long T is computationally expensive. Further, we want to keep T fixed to be 10 ms to facilitate comparison with prior examples. We thus utilize the following trick to improve computational efficiency that gives us an estimate of the shot noise contribution to $\mathrm{std}(\tau_{c})$ (i.e., $\mathrm{std}{\left(\tau_{c}\right)}_{\mathrm{shot}}$) that is distinct from the finite speckle sampling noise contribution to $\mathrm{std}(\tau_{c})$ (i.e., $\mathrm{std}{\left(\tau_{c}\right)}_{\text{speckle}}$). We can then get the total noise from the sum of the variances. Our trick is to sample the wave model for T=10  ms to get I(t). We then obtain $N_{\mathrm{avg}}=1000$ independent Poisson draws of this I(t) get 1000 instances of N(t). We obtain $g_{2}(\tau )$ for each instance of N(t) and average the 1000 correlation functions to get a more smooth $g_{2,\mathrm{avg}}(\tau )$. We then fit to get $\tau_{c,\mathrm{avg}}$ from $g_{2,\mathrm{avg}}(\tau )$. We repeat this process 100 times to obtain $\mathrm{std}(\tau_{c,\mathrm{avg}})$. Finally, we obtain $\mathrm{std}{\left(\tau_{c}\right)}_{\text{shot}}=\sqrt{N_{\mathrm{avg}}}*\mathrm{std}(\tau_{c,\mathrm{avg}})$, since shot noise contribution is proportional to $1/\sqrt{N_{\mathrm{avg}}}$.

**Fig. 7:**
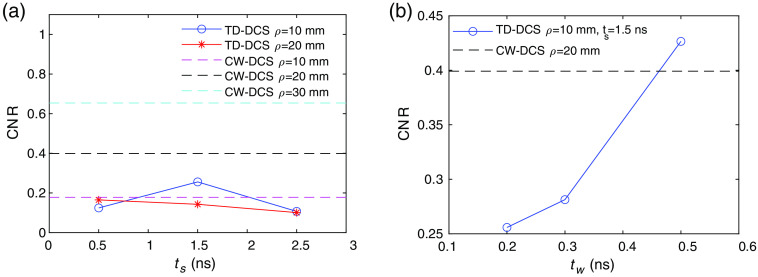
CNR comparisons of TD-DCS and CW-DCS in the shot noise limit. (a) CNR of TD-DCS as a function of $t_{s}$ at $t_{w}=0.2\;\mathrm{ns}$, ρ=10,20  mm, compared with CW-DCS at ρ=10,20,30  mm. (b) CNR of TD-DCS as a function of $t_{w}$ at $t_{s}=1.5\;\mathrm{ns}$, ρ=10  mm, compared with CW-DCS at ρ=20  mm.

With these photon flux, we then calculate the CNR for TD-DCS at ρ=10,20  mm with the effect of the IRF and coherence length taken into account, and CW-DCS at ρ=10,20,30  mm as shown in Fig. 7(a). We see that for TD-DCS, the maximum CNR for the cases we have calculated occurs at ρ=10  mm, $t_{s}=1.5\;\mathrm{ns}$, when $t_{w}$ is fixed to be 0.2 ns, but its CNR is still lower than CW-DCS at ρ=20  mm. The best performance for the cases we have simulated is CW-DCS at the largest source–detector separation of ρ=30  mm. Thus, when photon flux is taken into account, the relative performance of TD-DCS as compared to its CW-DCS counterpart has degraded. One way to increase the photon flux in TD-DCS is to increase the gate width $t_{w}$. We see that by increasing $t_{w}$, the CNR of TD-DCS at $t_{w}=0.5\;\mathrm{ns}$ has surpassed the performance of CW-DCS at ρ=20  mm. But at large $t_{w}$, the specificity to a particular photon pathlength L and thus the ability to differentiate tissue depths decreases.

**Table 1:** The photon flux used for all the simulation cases in Fig. 7.

	$N_{\text{photons}}$ received in MC	Photon flux (photons/s)
CW, ρ=10 mm	11,141,462	825,000
CW, ρ=20 mm	138,281	100,000
CW, ρ=30 mm	38,934	28,200
TD-DCS, ρ=20 mm, $t_{s}=0.5\;\mathrm{ns}$, $t_{w}=0.2\;\mathrm{ns}$	17,777	13,000
TD-DCS, ρ=20 mm, $t_{s}=1.5\;\mathrm{ns}$, $t_{w}=0.2\;\mathrm{ns}$	7428	5200
TD-DCS, ρ=20 mm, $t_{s}=2.5\;\mathrm{ns}$, $t_{w}=0.2\;\mathrm{ns}$	2862	2100
TD-DCS, ρ=10 mm, $t_{s}=0.5\;\mathrm{ns}$, $t_{w}=0.2\;\mathrm{ns}$	169,973	122,900
TD-DCS, ρ=10 mm, $t_{s}=1.5\;\mathrm{ns}$, $t_{w}=0.2\;\mathrm{ns}$	14,083	10,200
TD-DCS, ρ=10 mm, $t_{s}=2.5\;\mathrm{ns}$, $t_{w}=0.2\;\mathrm{ns}$	4242	3100
TD-DCS, ρ=10 mm, $t_{s}=1.5\;\mathrm{ns}$, $t_{w}=0.3\;\mathrm{ns}$	21,263	15,000
TD-DCS, ρ=10 mm, $t_{s}=1.5\;\mathrm{ns}$, $t_{w}=0.5\;\mathrm{ns}$	36,551	25,400

## 4 Discussion

We have developed a Monte Carlo wave model that is capable of simulating both TD-DCS and CW-DCS measurements from first principles. This model can be utilized to calculate the noise in TD-DCS and compare with CW-DCS measurements. We have validated our model with existing analytical expressions of $g_{2}(\tau )$ for CW-DCS, TD-DCS, and the noise model of CW-DCS. Our numerical model is capable of simulating any spectral profile, while existing theories always assume a Gaussian spectrum.^18^^,^^25^ Our system is capable of dealing with different brain activation geometries other than the two layer system we have specified in this manuscript. From the comparison of TD-DCS and CW-DCS, we have seen that the performance of TD-DCS is not necessarily better compared to CW-DCS, with the metric given by the CNR induced by tissue dynamics change in the deeper layer of a two layer sample, mainly due to the relatively low photon flux in TD-DCS since only a portion of the detected photons are selected. Methods that can increase the photon flux for a measurement gate, such as temporal focusing, could significantly improve the performance of TD-DCS, as we have seen that in the high photon flux limit, TD-DCS out-performs CW-DCS. The performance of TD-DCS is highly sensitive to the measurement parameters including gate delay time $t_{s}$, gate width $t_{w}$, and source–detector separation ρ. It also depends on the hardware properties including the wavelength and coherence length of the input laser light, the size of the detector, and the IRF profile. An analysis of the full parameter space is out of the scope of this manuscript. For a give TD-DCS measurement and brain activation geometry, the optimized parameters can be determined using the code of our Monte Carlo wave model. Our model will guide the future experimental design of novel TD-DCS systems.

## Code, Data, and Materials Availability

The Matlab code for the model is publicly available.^22^ Experimental conditions other than those reported in this manuscript can be achieved by varying the parameters in the model.
